# Supplementary material for: Engineering gene drive docking sites in a haplolethal locus in Anopheles gambiae
Source: Sci Rep. 2025 Oct 8;15:35074. doi: 10.1038/s41598-025-18484-y (PMC12508426; doi:10.1038/s41598-025-18484-y)
Supplement: Supplementary file 1 — Supplementary Material 1 [file 41598_2025_18484_MOESM1_ESM.docx]

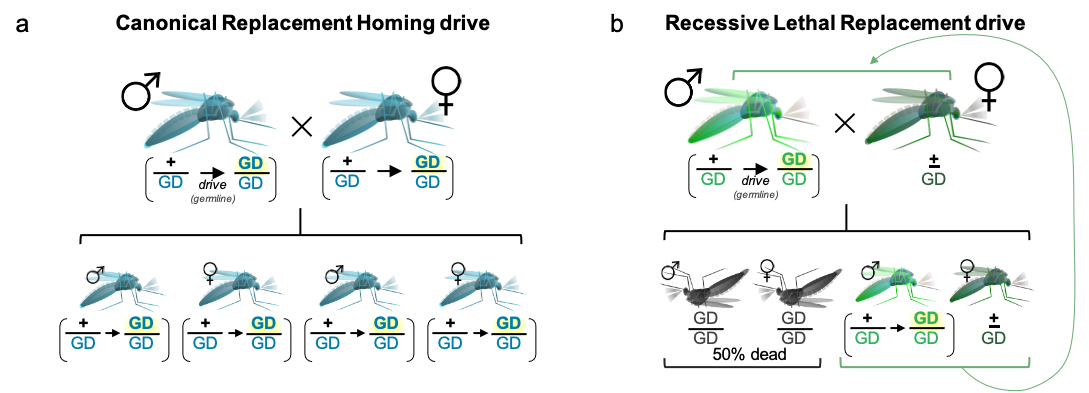


**Supplementary Figure 1. [a]** Canonical population replacement gene drives home in the germline of both sexes and permit survival of all individuals to facilitate population fixation of the drive allele and any associated cargo. dRP was originally designed to enable this type of population replacement drive. However this drive is not possible with the lines discussed herein due to unexpected Ribosome Minute phenotypes. **[b]** Late stages of recessive-lethal replacement (RLR) drive heterozygotes intercross, resulting in death of half the offspring and heterozygote dominant fixation of drive individuals. Drive occurs in the germline of a single sex (males here, bright green) contributing (up to) 100% drive-positive chromosomes to the next generation, while heterozygote survival is permitted by the contribution of a wild type chromosome from the non-driving sex (females here, dark green), which recapitulates the parental cross *ad infinitum* (curved green arrow). Concurrent death of homozyogtes (dark grey) each generation causes simultaneous population suppression by half.

**Supplementary Table S1. The full length plasmid sequence for dRP^pbnDonr^ sequence.** Different sequences for different features are described in the left column.

**Supplementary Table S2.** **The gDLT transgene sequence**. Different features and sequences are denoted in different colors and are described at left.

**Supplementary Table S3**. **gRNA tethered pairs.** gRNA L&M, N&O, P&Q, and R&S are tethered and full seuqnece is shown.

**Supplementary Table S4. Long ZPG promoter and 3’UTR.** The unpublished long ZPG promoter used to express in Cas9 in gene drive injection attempts

Expanded gels


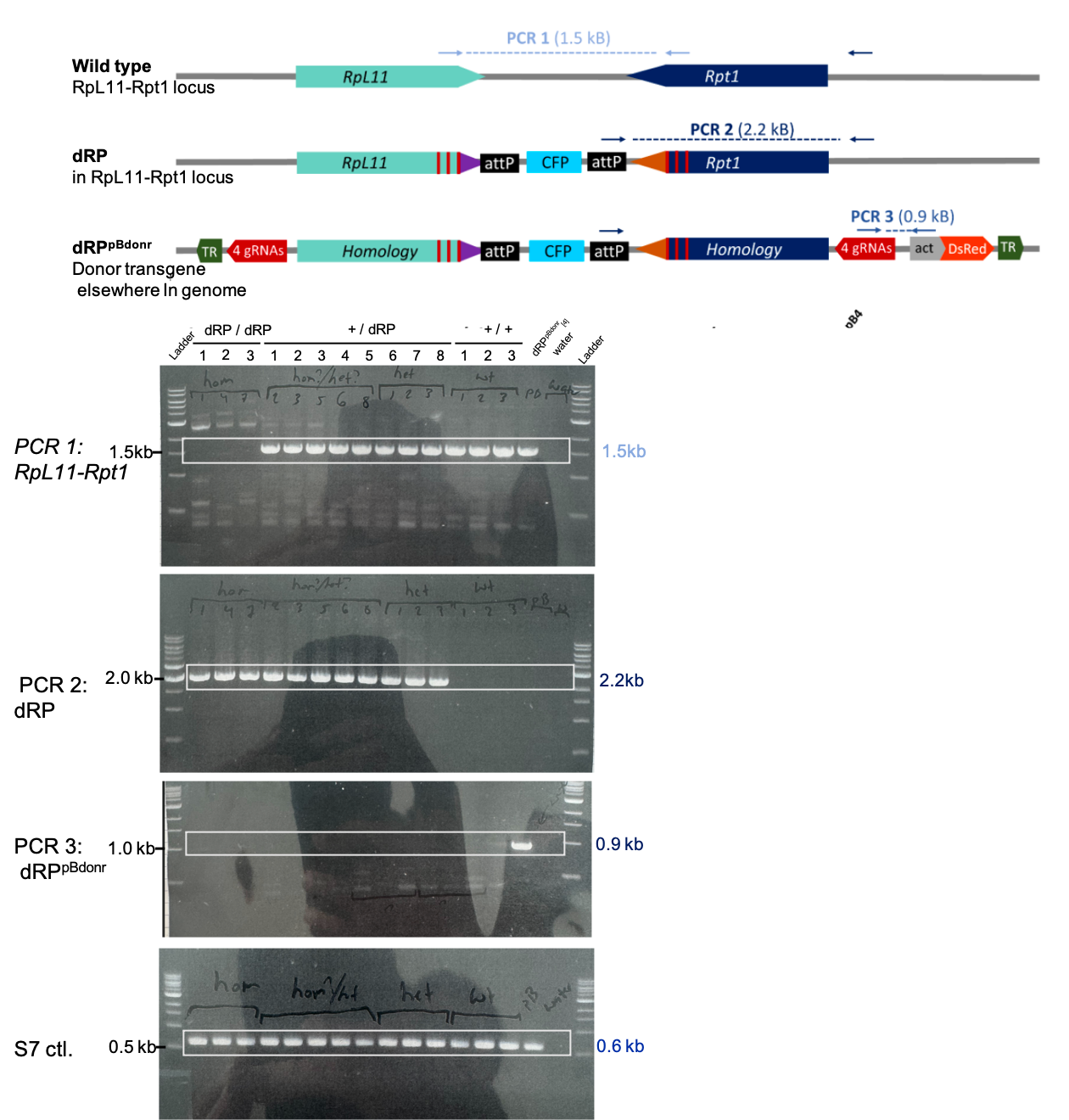


**Figure 1E expanded gel.** Samples tested are dRP/dRP homozygotes, +/dRP heterozygotes and +/+ WT controls. PCR 1; RpL11-Rpt1 PCR amplifies the WT endogenous *RpL11-Rpt1* sequence. PCR 2; dRP PCR amplifies the dRP insert. PCR 3; dRP^pBdonr^ PCR amplifies the donor transgene. S7 ctl PCR amplifies the S7 ribosomal control. Full-size gels are unavailable as all images were cropped during image acquisition. Gel images shown here are photographs of gels from within a lab notebook, not the original digital images; therefore contrast may differ from the gel shown in the main manuscript. Gels were cropped at the boxes for inclusion in the main manuscript.


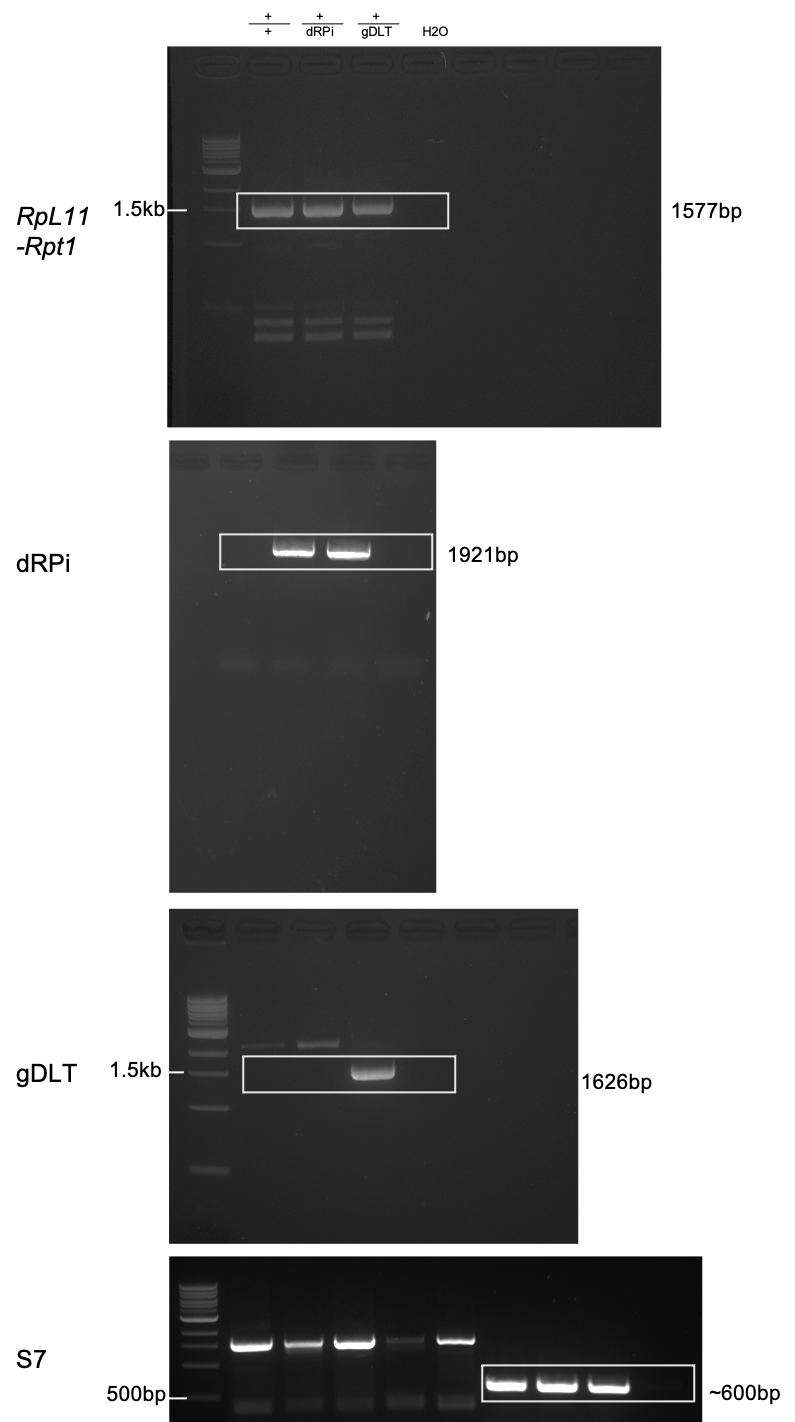


**Figure 2B expanded gels**. *RpL11-Rpt1* PCR amplifies the endogenous *RpL11-Rpt1* sequence in the above samples +/+, +/dRPi, +/gDLT and water controls. The RpL11-Rpt1 gel contrast has been modified compared to the gel image shown in the main manuscript to better show the ladder. dRPi PCR amplifies the dRPi insert. The ladder was cropped from the gel during image acquisition. gDLT PCR amplifies the gDLT transgene in dRPi. S7 PCR amplifies the S7 Ribosomal controls. The left five samples on this gel (S7) are irrelevant to the current study. Full-size gels are unavailable as all images were cropped during image acquisition. Gels were cropped at the boxes for inclusion in the main manuscript.


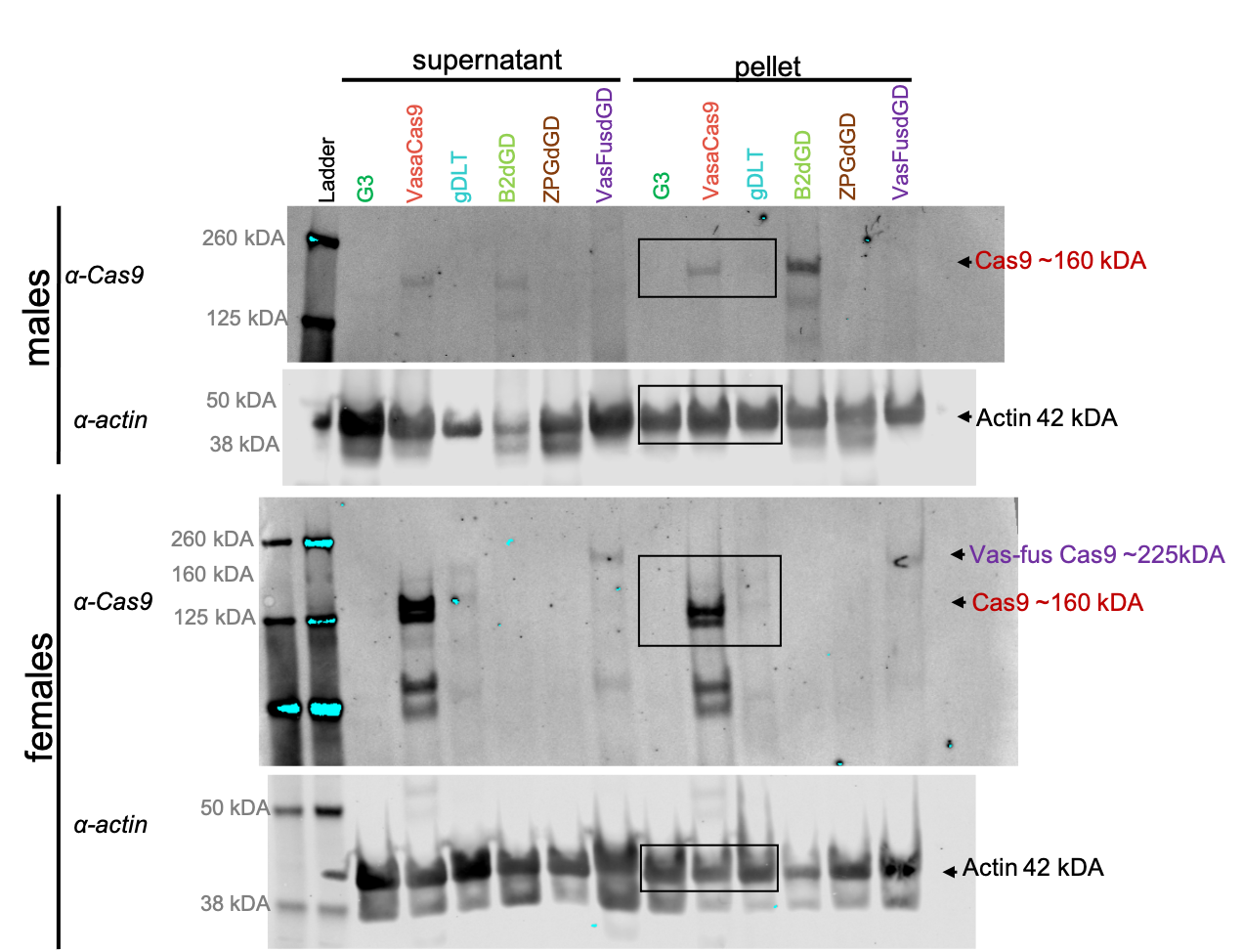


**Figure 2C expanded gel.** Cas9 is blotted for at approximately 160kDA however appears to run slightly faster than this in female samples. Actin is blotted at approximately 42 kDA. B2dGD, ZPGdGD and VasFusdGD samples are not otherwise discussed in this work. The Cas9 contained in VasFusGD is a fusion between Vasa protein and Cas9 protein and therefore runs at approximately 225 kDA. Full-size gels are unavailable as all images were cropped during image acquisition. Gels were cropped at the boxes for inclusion in the main manuscript.


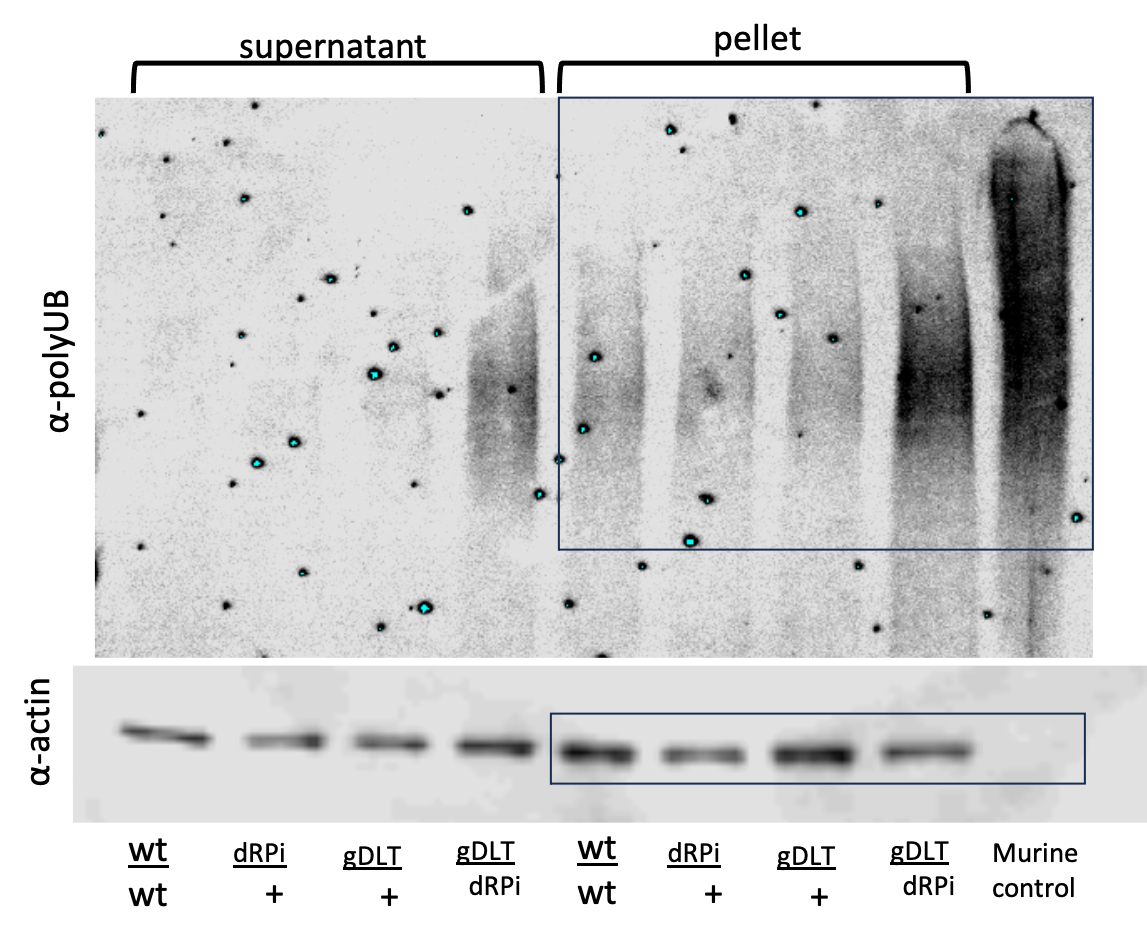


**Figure 2D expanded gel.** Polyubiquitin aggregates blotted for different genotypes; wt/wt, dRPi/+, gDLT/+, gDLT/dRPi . Supernatant samples are shown at left, pellet samples are shown at right. Murine positive control is shown at far right which does not blot for insect actin. Full-size gels are unavailable as all images were cropped during image acquisition. Gels were cropped at the boxes for inclusion in the main manuscript.


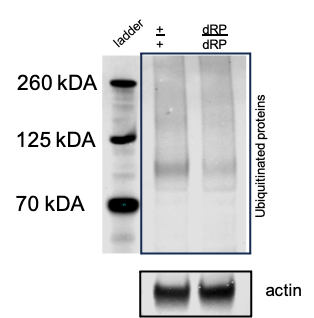


**Figure 3G expanded gel.** Polyubiquitin aggregates blotted for in wt and dRP/dRP homozygous samples. Ladder shown at left. Actin blotted at bottom. Full-size gels are unavailable as all images were cropped during image acquisition. Gels were cropped at the boxes for inclusion in the main manuscript.
